# Plant protection from virus: a review of different approaches

**DOI:** 10.3389/fpls.2023.1163270

**Published:** 2023-06-12

**Authors:** Irina Anikina, Aidana Kamarova, Kuralay Issayeva, Saltanat Issakhanova, Nazymgul Mustafayeva, Madina Insebayeva, Akmaral Mukhamedzhanova, Shujaul Mulk Khan, Zeeshan Ahmad, Linda Heejung Lho, Heesup Han, António Raposo

**Affiliations:** ^1^ Biotechnology Department, Toraighyrov University, Pavlodar, Kazakhstan; ^2^ Biology and Ecology Department, Toraighyrov University, Pavlodar, Kazakhstan; ^3^ Agrotechnology Department, Toraighyrov University, Pavlodar, Kazakhstan; ^4^ Department of Plant Sciences, Quaid-i-Azam University, Islamabad, Pakistan; ^5^ College of Business, Division of Tourism and Hotel Management, Cheongju University, Cheongju-si, Chungcheongbuk-do, Republic of Korea; ^6^ College of Hospitality and Tourism Management, Sejong University, Seoul, Republic of Korea; ^7^ CBIOS (Research Center for Biosciences and Health Technologies), Universidade Lusófona de Humanidades e Tecnologias, Lisboa, Portugal

**Keywords:** plant virus, resistance genes, PR-proteins, elicitors, method of apical meristems

## Abstract

This review analyzes methods for controlling plant viral infection. The high harmfulness of viral diseases and the peculiarities of viral pathogenesis impose special requirements regarding developing methods to prevent phytoviruses. The control of viral infection is complicated by the rapid evolution, variability of viruses, and the peculiarities of their pathogenesis. Viral infection in plants is a complex interdependent process. The creation of transgenic varieties has caused much hope in the fight against viral pathogens. The disadvantages of genetically engineered approaches include the fact that the resistance gained is often highly specific and short-lived, and there are bans in many countries on the use of transgenic varieties. Modern prevention methods, diagnosis, and recovery of planting material are at the forefront of the fight against viral infection. The main techniques used for the healing of virus-infected plants include the apical meristem method, which is combined with thermotherapy and chemotherapy. These methods represent a single biotechnological complex method of plant recovery from viruses *in vitro* culture. It widely uses this method for obtaining non-virus planting material for various crops. The disadvantages of the tissue culture-based method of health improvement include the possibility of self-clonal variations resulting from the long-term cultivation of plants under *in vitro* conditions. The possibilities of increasing plant resistance by stimulating their immune system have expanded, which results from the in-depth study of the molecular and genetic bases of plant resistance toward viruses and the investigation of the mechanisms of induction of protective reactions in the plant organism. The existing methods of phytovirus control are ambiguous and require additional research. Further study of the genetic, biochemical, and physiological features of viral pathogenesis and the development of a strategy to increase plant resistance to viruses will allow a new level of phytovirus infection control to be reached.

## 1 Introduction

Viruses cause various pathological changes, which affect all aspects of plant life. Most viral diseases are characterized by systemic damage, in which the virus moves from the primary site of inoculation to other parts of the plant organism (Ershova et al., 2022; Wang et al., 2022). A virus is usually present until it dies off once the plant is infected, and it is passed to the offspring by vegetative propagation. The viral infection manifests itself in the appearance of plants.

The physiology and biochemistry of the host cells and tissues have changed internally and as a result of the virus (Hull, 2014). The properties of the host plant, the virulence and aggressiveness of the virus strain, the length of the infection, and the environmental factors all affect the disease’s diagnostic indications, which can vary greatly (Jones, 2009). These factors also determine the duration of the incubation period. The incubation period is usually a few days or weeks, which can be more than a year for herbaceous plants. Viral, viroid, and mycoplasma diseases are considered very harmful due to their chronic nature. They damage plant species, which leads to plant stress, death, and low crop yields (**Table 1**). They also change the chemical composition and deteriorate the quality of tubers (Adolf et al., 2020; Kreuze et al., 2020). Viral infection significantly changes the metabolism of plants, which includes a reduction in the photosynthetic activity of plants, which suppresses carbohydrates and other types of metabolism (Anikina and Seitzhanova, 2015). Chloroplasts are destroyed, changed, or aggregated due to viral infection. This leads to the destruction of chlorophyll or its non-participation in synthesis. The degree of photosynthetic suppression depends mainly on the disease development, the characteristics of the virus strain, the disease development phase, the virus strain–disease development phase’s characteristics, the host plant, and the environmental conditions (**Table 2**) (Chen et al., 2019; Akbar et al., 2021).

**Table 1:** Reduction of crop yields under the influence of phytoviruses.

Crop	Decrease in productivity	Source
Sweet potato (*Ipomoea batatas*)	80%–98%	(Mwanga et al., 2001; Clark et al., 2012; Alam et al., 2013)
	30%–50%	(Silva and Fontes, 2022)
Tomatoes (*Solanum lycopersicum*)	42.1%–95.5%	(Hossain et al., 2011; Sevik and Arli-Sokmen, 2012; Farooq et al., 2021)
Okra (*Abelmoschus esculentus* L. Moench)	49%–84%	(Mishra et al., 2017)
	94%	(Dhankhar, 2016)
Potato (*Solanum tuberosum*)	10%–80%	(Mumford et al., 2016; Kreuze et al., 2020)
	10%	(Moses et al., 2017)
	10%–90%	(Salazar, 1996)
	15%–75%	(Gong et al., 2019)
Pepper (*Capsicum annuum*)	70%–80%	(Tolkach et al., 2019)
Watermelon (*Citrullus lanatus*)	10%–75%	(Xu et al., 2004; Tolkach et al., 2019)
Melon (*Cucumis melo* L.)	80%	(Sáez et al., 2022)
	30%–60	(Alonso-Prados et al., 1997)
Wheat (*Triticum*)	80%	(Perry et al., 2000)
	41%–63%	(Cisar et al., 1982)

**Table 2:** Type of overwhelming action under the influence of phytoviruses.

Type of suppression	Source
Plant growth delay	(Fletcher et al., 1998; Jin et al., 2016; Mumford et al., 2016)
Deterioration in the chemical composition and commodity qualities of the crop	(Alonso-Prados et al., 1997; Adolf et al., 2020; Kreuze et al., 2020)
Suppression of photosynthesis and carbohydrate metabolism	(Handford and Carr, 2007; Hull, 2014; Anikina and Seitzhanova, 2015; Chen et al., 2019; Akbar et al., 2021)
Suppression of the hormonal system	(Bari and Jones, 2009; Islam et al., 2019)
Violation of the water balance	(Hull, 2014; Anikina and Seitzhanova, 2015; Gupta N. et al., 2021)

Almost all viral diseases are characterized by a decreased total carbohydrate content (Handford and Carr, 2007). This occurs due to the destruction of chlorophyll in the mosaic and jaundice lesions. The disruption of the transport of photosynthetic products from the leaves to other plant organs is also affected by viral infections (Akbar et al., 2021). The outflow of starch is disrupted when the phloem is affected, which overloads the parenchyma cells. As a result, the leaves become thicker, leathery, and brittle (Yu, 2015). Changes in the permeability of the parenchyma cell cytoplasm or the carbohydrase activity may also cause delayed starch outflow from the leaves. The study of the histological structure of plants revealed hypertrophy and hyperplasia, which resulted in the formation of tumors and enations. Many viruses affect the vascular system xylem and phloem, which causes the formation of tillers and cell death (Hull, 2014). As a result, the wilting of plants, the delayed outflow of the assimilates from the leaves, and the appearance of necroses on the vegetative plant in tubers and fruits are observed (Gupta N. et al., 2021). The phenomena of hypoplasia and metaplasia accompany the dwarfism of plants and the color changes in the case of virus infection. Many viruses cause changes in the fine structure of infected cells. Vesicles are formed at the periphery of the chloroplasts when they are under the influence of thymoviruses. Chloroplasts in tobacco mosaic virus (TMV)-infected tobacco cells become deformed and often degenerate. The formation of new chloroplasts is also inhibited in these types of cells (Bhattacharyya et al., 2015). These changes are responsible for the chlorosis and mosaic coloration of the affected leaves.

Viral infection causes metabolic abnormalities in the cells of the diseased plant. For example, the water regime is disturbed when it is infected with phytoviruses, which are accompanied by changes in the transpiration intensity and the water inflow due to changes in the vascular system (Anikina and Seitzhanova, 2015). The transpiration flow slows down, and the intensity of transpiration decreases, resulting from changes in the conductive system. In addition, there are changes in leaf transpiration surface due to the development of necroses (stomata malfunction) and the death of a part of the leaf apparatus. The metabolism of the affected plant changes, which can lead to its death due to the water balance disruption (Gupta N. et al., 2021). Almost all viral diseases are characterized by the disruption of nitrogen metabolism. Viruses have no enzymatic activity, but the essential role of the host plant’s enzymes is observed with the changes in the nitrogen-containing compounds (Hull, 2014). The proteolytic activity is significantly increased in potato leaves that are affected by the wrinkle mosaic, which can only be attributed to the proteinases of the potato itself. An increased soluble and nitrate nitrogen content is observed in the leaves and tubers of the potatoes that are affected by leaf curl, which is associated with the disruption of the nitrate restoration and protein synthesis processes. The total amount of nitrogen in the plant does not change when tobacco is infected with a mosaic pathogen. A significant portion of it still goes to virion building since the viral protein is formed at the expense of the host plant’s protein. The amount of non-protein nitrogen in infected tobacco is greatly reduced under nitrogen deficiency conditions. In contrast, the content of free amino acids increases with excess nitrogen probably due to the increased hydrolysis of the plant proteins (Dyakov et al., 2007).

Studies about the respiration of plants affected by viruses showed that viral infection stimulates the activity of dehydrases, which affect the initial phases of respiration, and the peroxidase activity of the affected plants increases at the same time (Hull, 2014). An increase in the respiration activity in tobacco plants during TMV infection was observed, whereas an increased oxidative activity after the influence of the beets and potatoes’ viral infection was also revealed. The activation of respiration is attributed to the protective reactions of the host plant. The peak of the respiration activity and the oxidative enzymes inhibit the reproduction of the virus at the beginning of the infection. The respiration intensity of the infected plant decreases, and the virion synthesis is activated. Many researchers show that viral diseases of plants are accompanied by dwarfism; the appearance of tumors and enations; changes in the shape of the leaves, flowers, and fruits; the formation of excessive buds; and the imbalance of the hormonal metabolism in plants under the influence of viral infection (Ma et al., 2022; Wang et al., 2022). This review aims to analyze and discuss contemporary methods for controlling viral infestation in plants by comparing various methods and taking into account the negative effects of viral diseases as well as the unique difficulties associated with viral pathogenesis. It also aims to identify efficient preventative measures to deal with phytoviruses by examining the molecular and genetic principles underpinning viral pathogenicity. As it focuses on creating strategies to control viral infections in plants, which is crucial for guaranteeing good crop production and addressing issues with food security, this evaluation helps to achieve Sustainable Development Goal 2: Zero Hunger by addressing these objectives.

## 2 Biotechnological approaches to countering phytoviruses

### 2.1 Creation of varieties resistant to viruses based on the study of molecular stability mechanisms

The fight against viral infection is complicated due to the rapid evolution of viruses and the obligate parasitic nature of viruses. They penetrate the nucleus of cells, where viruses use the internal reserves of the plant for their reproduction (Tian and Valkonen, 2013; Jones and Naidu, 2019). Promising measures against plant viruses include breeding programs in regard to creating virus-resistant forms, which are in particular based on the marker-assisted selection method to evaluate the genetic defenses of a variety as well as the creation of transgenic varieties with high resistances to viral diseases (Klimenko et al., 2019; Akhter et al., 2021). It has become possible to insert certain genes into the genome of a plant, which is based on genetic engineering, which has resulted in new protective proteins that determine that the resistance to viruses is synthesized in the plant cells. Plant varieties with genes of extreme resistance to certain viruses have already been obtained. For example, a variety of soybean L29 with exceptional resistance to isolates of G5, G6, G7, and G5H strains has been obtained (Tran et al., 2018). R-resistance genes are expressed in plant cells during infection. More than 200 R genes with plant resistance to viruses, bacteria, and fungi have been cloned (Sharipova et al., 2013). Potato R genes are responsible for plant resistance to the potato virus X (PVX). Tomato SW-5 gene resists tomato spotted wilt virus, and tomato Tm and Tm2 genes resist tomato mosaic virus. Mutational analysis showed that R genes encode translation initiation factors that lead to overcoming the RNA virus infection (Sharipova et al., 2013). Studies about the molecular mechanisms of extreme resistance and their relationship with hypersensitive response concluded that the same NLR genes can trigger both extreme resistance and hypersensitive response. Genes that generally provide the phenotype of extreme resistance can be stimulated in order to induce a hypersensitive response by experimentally increasing cellular levels of derived pathogen proteins (Ross et al., 2021).

Most plant NLR proteins consist of three primary domains, which include the N-terminal helix-helix domain, the Toll/interleukin-1 receptor or divergent helix-helix domain, the central nucleotide-binding domain, and the C-terminal domain rich in leucine repeats (LRR). These proteins function as intracellular immune receptors, and the nucleotide-binding state partially controls their ability to induce an immune response. Inactive NLR proteins are specifically bound to ADP, whereas the recognition and binding of the effector pathogen protein allow the NLR to switch to an active and ATP-bound state that can initiate an immune response (Lolle et al., 2020). A total of 30 genes that are responsible for the resistance of potato plants to viruses X, T, A, and M have currently been identified. Genetic silencing is the deactivation or reduction of one of the plant’s genes using RNA interference, which involves the suppression of the gene expression by double-stranded RNA and is also used in order to create genetically modified virus-resistant plants. RNA silencing is an essential antiviral mechanism (Li and Wang, 2022; Lu et al., 2022). A DNA fragment is isolated from the genome and placed in a genetic construct in an inverted (antisense) position to turn off the target gene. Synthesized RNA does not encode anything in this case, but it can bind to the target gene’s mRNA. Translation stops and mRNA destruction occurs, and the expression of the target gene is drastically reduced or even completely stopped (Calil and Fontes, 2017; Kochetov and Shumny, 2017). There are currently great expectations for studying the regulatory pathways of plant resistance formation. Signal transduction from external factors leads to the activation of serine/threonine protein kinases, which phosphorylate the threonine or tyrosine residues of other regulatory proteins in this group that is activated by phosphorylation. The coordinated interaction of regulatory signaling pathways occurs during the plant infection, which results in the expression of resistance genes and increased plant defense against pathogens. A study of plant antiviral defense mechanisms revealed that when attacked by an infection, they activate the genes encoding PR proteins, which are pathogenesis-related proteins.

The type and level of PR-protein accumulation depend on the nature and level of the plant damage. Some PR proteins, such as proteinases and β-1,3-glucanases, promote the virus infection of plants. Other PR proteins, such as proteinase inhibitors, ribonucleases, and peroxidases, effectively protect plants against viruses. Signaling systems regulate their coordinated accumulation in plants. Thus, it has been determined that the accumulation of PR-10 proteins around the pathogen introduction or wounding site indicates their participation in plant defense mechanisms (Prasad and Srivastava, 2017). Transgenic plants with increased RNase expression are more resistant to pathogens than the original plants (Kochetov and Shumny, 2017). The disadvantages of the genetically engineered approaches include that the acquired resistance is often specific and short-lived, and there is a *gene silencing* problem (Li and Wang, 2022). The negative factors of cultivating genetically modified plants resistant to certain viruses include the possible redistribution of the virus species structure, which can spread other harmful viral infections. There are restrictions on using genetically modified organisms (GMOs), such as genetically modified potato varieties not being used in Kazakhstan. Modern methods of prevention, as well as the diagnosis and recovery of planting material, are at the forefront of potato virus infection control (Mumford et al., 2016; Kesiraju and Sreevathsa, 2017; Wang et al., 2022).

### 2.2 Use of genome editing to combat virus infection

Progress in the development of resistant varieties, including those based on transgenic methods, is slow due to the lack of host genes and the duration of the breeding process and the problems associated with the limited use of transgenic plants in many countries (Tiwari et al., 2022). Genome editing technology is a promising approach to control viral pathologies. Breakthroughs in genome editing technology have been made in functional genomics and crop improvement (Zhang et al., 2018; Hofvander et al., 2022). Zinc finger nucleases (ZFNs) and effector nucleases (TALEN) were used as the first available genome-editing tools. These nucleases are chimeric proteins created by fusing their respective DNA-binding domains (DBDs) with the DNA cleavage domain of Fok I restrictase. The method of genome editing based on ZFNs and TALENS has been used for several plant species despite being labor-intensive, and important achievements have been obtained (Shan et al., 2013). However, nowadays, a new genome editing platform has been successfully used, which has surpassed the efficiency of ZFNs and TALEN in plants. According to scientists, CRISPR/Cas is currently the most powerful biological tool for targeted genome modification (Silva and Fontes, 2022; Tiwari et al., 2022). Many researchers consider the CRISPR/Cas-mediated immunity mechanism based on regularly spaced short palindromic repeats (CRISPR) and CRISPR-associated (Cas) proteins crucial in the fight against pathogens. This type of immunity has evolved as an adaptive immune system in archaea and bacteria against an invasion of foreign nucleic acids from viral or plasmid pathogens (Nussenzweig and Marraffini, 2020; Nidhi et al., 2021).

In recent years, there has been growing interest in using various aspects of CRISPR/Cas technologies to create plants resistant to viral pathogens. There are two ways to use CRISPR/Cas systems: the first involves direct targeting of viral genomes, and the second involves introducing targeted mutations into specific host plant genes that encode proteins used by the virus for successful replication and spread in the plant. The first method is the most widespread (Kavuri et al., 2022). Cas class 1 system include types I, III, and IV with different variants and effector complexes, whereas Cas class 2 systems comprise types II, V, and VI with an effector module containing a single multifunctional protein (Kavuri et al., 2022). Because of their simpler organization, class 2 Cas systems are most widely used as genome-editing tools, with type II and V systems using Cas9 and Cas12 enzymes to edit DNA. Notably, the CRISPR/Cas9 type II class 2 protein is one of the first Cas proteins to be studied, leading to its widespread use for DNA editing in animals, plants, and bacteria (Kavuri et al., 2022). Since the discovery of the CRISPR/Cas9 system in *Streptococcus pyogenes*, related systems have been discovered in many bacterial and archaeal species (Robertson et al., 2022). CRISPR/Cas9 has now produced important advances in plant research because of its simplicity, multiplexing, cost-effectiveness, high efficiency, and minimal target bias. The practical application of the CRISPR/Cas technology faces several bottlenecks whose solution is crucial for plant gene editing, one of which, for example, is the method of delivering components of the Cas9 RNA editing complex into plants. Gene delivery using plasmid DNA and *Agrobacterium* transformation (the main method used) leads to transgenic plants, which is prohibited by the legislation of many countries. The preferred methods are direct (free-associated) delivery of kgRNA and Cas9 protein, which have been actively developed recently (Tiwari et al., 2022). Knowledge of all aspects of CRISPR/Cas systems is constantly expanding.

There are certain limitations in DNA editing using CRISPR, such as the protospacer adjacent motif (PAM) site requirement, non-targeted mutations, and low efficacy against viruses (Ahmad et al., 2020). The recently identified CRISPR/Cas type VI systems can overcome some of these limitations, which use the Cas13 protein to provide sequence-specific cleavage of single-stranded RNA (ssRNA) molecules (Wolter and Puchta, 2018; Kavuri et al., 2022). The discovery of Cas endonucleases targeting RNA molecules marked a new turn in developing CRISPR/Cas technologies. One of the best known is the Cas13 class 2, type VI protein (including Cas13a and Cas13b). Unlike most CRISPR/Cas systems, Cas13 lacks a DNAase motif but contains two RNAase domains (HEPN). RNA manipulations are advantageous over DNA editing because they prevent unwanted pleiotropic effects, and RNA products can be precisely and spatially regulated (Robertson et al., 2022; Sharma et al., 2022). According to the Abudayyeh et al. research, Cas13 cleaves only target RNA molecules and can cleave ssRNA molecules containing sites homologous to cRNA, while, like Cas12a, Cas13 does not need tracrRNA and depends only on cRNA (Abudayyeh et al., 2017). Since most plant viruses contain RNAi genomes, this research opens up new technological possibilities for combating plant viruses. Another widely used RNA editing nuclease is FnCas9 from *Francisella novicida* (Price et al., 2015). This enzyme also works with guide 23 RNA and targets endogenous RNA *in vivo*. However, unlike Cas13, this protein does not contain HEPN domains. It has been suggested that FnCas9 may either recruit endogenous RNAases to cleave the target RNA or possess alternative domains with endonucleolytic activity (Robertson et al., 2022). Like Cas13, FnCas9 has been reprogrammed to target and inhibit RNA-containing human and plant viruses by blocking viral RNA translation and replication (Price et al., 2015; Zhang et al., 2018). Most research on the direct use of the CRISPR/Cas system has been performed on viruses belonging to the family *Geminiviridae*, which cause significant yield losses in economically important crops. The first experiments on CRISPR-mediated resistance to geminiviruses were carried out on the beet severe curly top virus (BSCTV, genus *Curtovirus*) and the bean yellow dwarf virus (BeYDV; genus *Mastervirus*) in *Nicotiana benthamiana* and *Arabidopsis thaliana* plants (Baltes et al., 2014). CRISPR/Cas9 constructs (including kgRNA and Cas9) were targeted to cut coding (such as the replication-related protein gene [Rep]) and non-coding regions in viral genomes (such as Rep binding sites) and an invariant non-coding sequence, the nanonucleotide [TAATATTAC], common to all geminiviruses, contained in the intergenic region [IR]. Plants were transfected with the obtained constructs, and it was shown that the transgenic plants showed a high level of resistance to the target virus, which is manifested by a decrease (up to 87%) in virus accumulation and a reduction of symptom manifestation (Baltes et al., 2014). Zhan et al. showed that the CRISPR/Cas13a system effectively provides a wide range of resistance in transgenic potato plants to potato virus Y (PVY) strains (Zhan et al., 2019). Also, studies by Zhao showed that the LshCas13a system can degrade viral RNA genomes and confer resistance to the RNA virus in monocotyledonous grain plants (Zhao et al., 2020). Transgenic rice plants carrying the CRISPR/Cas13a system were created with three sgRNAs, each targeting the RNA genomes of southern rice black-streaked dwarf virus (SRBSDV) and rice stripe mosaic virus (RSMV). The study confirmed the suppression of viral infection in transgenic rice plants, indicating that CRISPR/Cas13a can also effectively target viral RNA in monocotyledonous plants.

The advantages of this technology include that by working precisely at the gene level, it is possible to bypass the problems of “genetic modification” because genome editing occurs without integrating foreign DNA or RNA into the host genome. Moreover, this method is simple and versatile when compared to other modern breeding technologies (Robertson et al., 2022). Unlike genetically modified organisms, CRISPR/Cas changes the existing genome without introducing foreign genes, particularly site-directed nucleases (SDN1 and SDN2). Consequently, varieties obtained using CRISPR/Cas are expected to be transgenic-free, and biosafety issues will be eliminated. The creation of next-generation systems characterizes the current stage of development of CRISPR methods such as CRISPR-MAD7 and CAS12A nucleases, increasing accuracy, range of possibilities, and applications. The efficiency of the CRISPR-MAD7 system has been proven on mutant rice and wheat plants at 65.6% (Silva and Fontes, 2022). Using MAD7 expands the CRISPR genome-editing toolkit because of its highly efficient target for gene disruption and insertion. According to (Silva and Fontes, 2022), using the new CRISPR/CAS systems (CAS3, CAS12, CAS13, and CAS14) as multiplexed SGRNAs by targeting them to different sites is a new and more effective strategy for increasing resistance to a wide range of viral infections and disease control in the field.

### 2.3 Culture of apical meristems to eliminate the virus

One of the main methods used for recovering virus-infected plants is the method of apical meristems. This method uses the apical virus-free zone to obtain an initial healthy plant, which serves as the progenitor of the starting material for primary potato seed production. The effectiveness of this method has been repeatedly confirmed for many plant crops (Bi et al., 2018; Zhang et al., 2019). Many valuable potato varieties have been renewed and used in production for a long time. Potato yields have increased by more than 42% with the implementation of this method (Galeev et al., 2018). An apical meristem is a group of meristematic (formative) cells organized into a growth center, which occupy the terminal position in a shoot or root and form all organs and primary tissues. The upper part of the apical meristem is represented by initials, which are a single cell in horsetails, many ferns, and a multicellular structure in seed plants. The nearest derivatives of the initial cells are often distinguished in the protomeristem zone. There are currently several hypotheses about the reasons for the absence of viral infection in the apical meristem, which are provided below.

1. The absence of a conductive system in the apex slows the spread of viruses from cell to cell. The growth of the apical meristem is faster than viral propagation.
2. The high concentration of auxins in the apex excludes the possibility of virus replication.
3. There are mechanical barriers to the viral infection advancement into the meristematic zone due to the small size of the plasmodesmata.

The apical meristems of the seedlings are isolated at 12–13 plastochrone, which is the time interval between the initiations of two leaf tubercles. Isolated meristems are cultured under aseptic conditions on nutrient media rich in macro-salts and micro-salts with an increased concentration of cytokinins (6-BAP 2 mg/L). The temperature was maintained at 25°C ± 2°C in an air-conditioned culture room, which included 70% humidity, 5 klx illumination, and a photoperiod of 16 h. A small part of the 0.15- to 0.5-mm meristem is usually planted on the nutrient media. The general pattern is provided next. The smaller the size of the meristem, the more likely virus-free plants are obtained. It is isolated under sterile conditions of a laminar box under the magnification of a binocular microscope. On average, 30–45 days pass from planting the meristem on a medium to forming seedlings with five to six leaves, which sometimes takes 2 to 8 months. The media are renewed as they are depleted, and the seedlings are periodically transplanted to new media under sterile conditions. The disadvantages of this method include the difficulty of obtaining the initial virus-free material. The peculiarities of the apical meristem method consist of the complexity of the regeneration of meristems that are 0.1 mm in size. Their engraftment and regenerative ability increase when the size of the meristems increases, but the risk of viruses being present also increases (Moses et al., 2017). It is necessary at this stage to increase the method’s effectiveness and suppress the viral pathogenesis in a larger area of the meristem. According to some experts, it is also advisable to use additional procedures, such as treatment with UHF rays with a narrow-band laser as well as thermotherapy, cryotherapy, and chemotherapy (Zhao et al., 2018; Wang et al., 2021; Bettoni et al., 2022).

### 2.4 Thermotherapy method

The thermotherapy method, which includes heating without lighting, was conducted on the potato tubers and microplants. Exposure modes may vary depending on the varietal characteristics. The efficiency of rehabilitation during the thermal treatment of the potato microplants is 2.4 times higher than for the same method for tubers (Oves and Gaytova, 2016). According to the research by Wang et al. (2021), *in vitro* cultured shallot shoots infected with onion yellow dwarf virus (OYDV) and shallot latent virus (SLV) were thermo-treated at a constant temperature of 36°C for 0, 2, and 4 weeks. The meristems (0.5 mm) that contain one to two leaf primordia were then excised and cultured for shoot regrowth. The meristem culture with thermotherapy produced much higher virus-free plants, which included 70% for OYDV, 80% for SLV, and 50% for both viruses (Wang et al., 2021). Bi et al. (2018) described a droplet-vitrification cryotherapy method for eradicating grapevine leafroll-associated virus-3 (GLRaV-3) from *Vitis* plants’ diseased *in vitro* shoots. All the plants recovered after cryotherapy and were free of GLRaV-3 in two wines, which included one table and one rootstock cultivar *Vitis* spp (Bi et al., 2018).

### 2.5 Method of chemotherapy

The chemotherapy method is based on adding broad-spectrum antiviral drugs, such as virazole, to the nutrient medium where the plant explants are cultivated. The experimental results showed the effectiveness of virazole and amixin for inhibition (78.2%). The high antiviral activity of this drug was found in different cultures, such as against *Odonotoglossum cymbidium* ringspot virus and rose mosaic viruses, but its pronounced toxic effect, which inhibits the differentiation and proliferation processes in plant tissue culture *in vitro*, was also revealed. Substances that are capable of inactivating several viruses have been identified. Particular success in this direction was obtained on potatoes, tobacco, tomatoes, narcissus, and tulips (Sochacki and Podwyszyńska, 2012; Bettoni et al., 2022). Ryabtseva et al. (2015) discovered that for the recovery of the potatoes’ *in vitro* culture, the following virus inhibitors showed a high efficiency, which included chitosan with a dose between 0.1% and 0.01%, interferon with a dose between 0.05% and 0.1%, and virazole with a dose of 0.01%. The percentage of the plants that recovered from viruses was from 25% to 100%, which depended on the variety (Ryabtseva et al., 2015).

The yield of healthy plant regenerants compared to the control was higher by 14.3%–50.0% when antiviral medications, such as interferon, Kagocel, and Arbidol, were used in the nutrient medium in the amount of 50 mg/L. This was revealed in a study on the effect of antiviral drugs in *in vitro* culture on the yield of viable plant explants (Yalovik et al., 2019). Virus inhibitors include chemical nature malonic, oxalic, ascorbic, nucleic acids, antibiotics, gibberellin, heteroauxin, malachite green dye, methylene blue dye, safronin dye, alkalis, formaldehyde, urea, and the salts of heavy metals. The inhibitors of plant viruses were also found in the leaves of currants, forest strawberries, raspberries, pelargonium, parsley, wormwood, apples, cherries, maple, linden, and beets (Anikina and Seitzhanova, 2015). Several groups of synthetic antiviral compounds have also been identified. Most of them are analogs of bases and nucleosides, and their metabolites, such as the purine base derivative 8-azaguanine, which inhibits TMV, PVX, PVY, and 1-β-d-ribofuranosyl-1,2,4-triazole-3-carboxamide (ribavirin), are also active against numerous plant viruses, such as TMV, PVX, and CMV. Their action is based on the inhibition of viral genome replication. Representatives of pyrimidine-like compounds include 2-thiouracil and 5-azadihydrouracil. They inhibit the reproduction of viruses TMV, PVX, PVY, and CMV by inhibiting the biosynthesis of uridine-5-phosphate from orotidine-5′-phosphate by decarboxylase inhibition (Kumar et al., 2001; Sharipova et al., 2013).

The effectiveness of virocides has also been proven with outdoor plant treatments. The limiting factor is the phytotoxicity of virocides and the teratogenic effect of some virocides (Maksimov et al., 2019). In addition, there are still questions about the mutagenic effect of antiviral drugs, and their use also requires control of the identity of the material (Ryabtseva et al., 2015). Both thermotherapy and chemotherapy methods are used as auxiliary tools of the apical meristem method in *in vitro* culture. These methods essentially represent a single biotechnological complex method of plant virus recovery. This method is widely used worldwide, and different plant species have been revitalized with its help (Alam et al., 2013; Moses et al., 2017; Wang et al., 2021). Significant disadvantages of the tissue culture-based rehabilitation method include the possibility of self-clonal variations. These types of plants can deviate from the original variety with their morphological traits and properties, which means that they lose varietal individuality. This is a serious risk for primary seed production because the valuable economic traits of the cultivated variety are still under consideration, and they obtain mutants that they may not have (Oves and Gaytova, 2016; Antonova et al., 2017; Kesiraju and Sreevathsa, 2017). The researchers concluded that regular control of the material for identity is required during the long-term cultivation of plants in the tissue culture. The search for a solution to this problem has led some researchers to propose complementing the apical meristem method with the clonal selection or verifying the genetic identity of the regenerated plants *in vitro* using random amplification of polymorphic DNA (RAPD) (Evstratova et al., 2018). In addition, the cured potatoes can quickly be reinfected in the field.

## 3 Methods of combating viruses in the field

Virus reservoirs can be weeds along the field edges, birds and insects that travel long distances, and viruses, which can also be transmitted *via* service aggregates. Preventive measures, such as spatial isolation and insecticidal and stimulant treatments, are essential to controlling the spread of plant viruses (Tian and Valkonen, 2013; Wasilewska-Nascimento et al., 2020). These medicines have become important economic factors in regard to increasing the profitability and eco-friendliness of production. They have a beneficial effect on plant product growth, development, yield, and quality, but they are also inducers of resistance to abiotic stress factors and various phytopathogens, which include viruses (Palukaitis et al., 2017). The research for biosafe means of controlling phytopathogens is relevant in light of the increased attention to the ecologization of agricultural crop production. The search for opportunities to stimulate the immune system of plants and build a plant protection system based on pesticide application as well as on the reserve possibilities of the organism itself is essential, which open up new prospects for the development of biotechnology (Maksimov et al., 2019).

Nucleotide-binding proteins with leucine-rich repeats (NLRs), which recognize intracellular proteins of pathogenic origin, often control immune responses to pathogens in plant organisms. Genetic resistance to plant viruses is often phenotypically characterized by programmed cell death at or near the site of infection, which is a hypersensitive reaction. New approaches in order to control viral pathogens are urgently needed (Akhter et al., 2021). One type of approach is plant immunization, which is a process of activating the body’s natural defense systems. The possibilities of increasing plant resistance by stimulating their immune system have expanded, which results from the in-depth study of the molecular and genetic bases of plant resistance to viruses and the investigation of the mechanisms of induction of the defense reactions in the plant organism. The key role in the induction of immunity belongs to different substances, which are called elicitors.

The biological immunization of plants involves treating them with weakened cultures of pathogens, non-pathogens, or their metabolites (Kothari and Patel, 2004; Dyakov et al., 2007). Chemical immunization is based on using substances called elicitors, resistance inducers, activators, or immunomodulators, which activate the defense reactions. A cascade of defense reactions is triggered in plants under the action of these drugs, and chemical and physical barriers are formed that prevent pathogen development. Inducers typically send excitatory signals to plant defense genes, which activate a cascade of defense responses and ultimately induced systemic resistance (Calil and Fontes, 2017; Beris et al., 2018). Elicitors were associated with the induction of phytoalexin synthesis at the beginning of these types of studies, but further studies revealed other protective responses of plants that expanded the list of compounds under the influence of how plants trigger their defense mechanisms (Poliksenova, 2009). Biologically active substances have different characteristics, and many of them are plant growth regulators, which act as elicitors.

Zircon is among the medicines with high elicitor activity, which is obtained based on the plant material of *Echinacea purpurea*. Zircon’s efficiency has been proven both as a growth stimulator and as a resistance inducer against various types of diseases, which include viral diseases (Malevannay, 2001). The zircon phytoregulator exhibited an antiviral effect on potato *in vitro* plants when introduced into a Murashige and Skoog culture medium. The application of a zircon phytoregulator at a dose of 0.25 ml/L on potato *in vitro* plants reduced the development of the PVY virus by 10%–70%, which depended on the variety. We show that chlormequat chloride in the Murashige and Skoog culture medium at a dose of 0.3 ml/L exhibited antiviral activity when the antiviral action of the growth regulator chlormequat chloride (C_5_H_13_Cl_2_N) was analyzed, which belongs to the group of retardants. The percentage of regenerants that are affected by the potato Y virus decreased from 14% to 37.5%, which depended on the variety (Anikina and Seitzhanova, 2015).

The study of the action of plant polyphenols and flavonoids revealed their high effectiveness in suppressing plant virus infection, particularly with the tobacco mosaic virus, the apple stem borer virus, the tomato spot nepovirus, and the potato X-virus (Chojnacka et al., 2021). Evstratova et al. (2018) showed the high elicitor activity of chitosan-based medicine against the potato Y virus (Evstratova et al., 2018). The search for biologically active substances (BASs) that are capable of stimulating plant immune system mechanisms has significantly increased (Prasad and Srivastava, 2017; Gupta AK. et al., 2021). Mandal (2010) investigated the resistance of eggplant under the action of four elicitors, which included salicylic acid, chitosan, methyl salicylate, and methyl jasmonate. The effect of the elicitors resulted in a threefold to fivefold increase in the accumulation of phenols and lignin in the tissues in addition to an increase in the activity level of the main protective enzymes phenylalanine ammonia-lyase peroxidase, polyphenol oxidase, cinnamic alcohol dehydrogenase, and catalase several times, which certainly contribute to the plant’s resistance to pathogens (Mandal, 2010). The effectiveness of the steroid glycoside compounds against the tobacco mosaic virus has in particular been illustrated. It was discovered that steroidal glycosides act comprehensively, and under their action *in vitro*, the infectivity of the virus is reduced, and the particle structure and antigenic properties are not violated. They have an elicitor effect on the host plant metabolism. At the same time, RNAase is activated, the formation of new proteins is induced, and the cell ultrastructure is stabilized. Other researchers also confirmed the effectiveness of steroidal glycosides as immunomodulators that increase plant resistance to phytopathogenic viruses (Poliksenova, 2009).

The use of microbial inoculants to induce systemic resistance against viral diseases is of great interest. The researchers showed the high bioprotective potential of microbial inoculants, which included *Pseudomonas fluorescens* and *Bacillus* sp. isolated from banana roots, against the banana virus disease, which was caused by the banana bunchy top virus (BBTV). The banana plants were inoculated with *P. fluorescens* Pf1 and CHA0 strains in combination with endophytic bacterial strains EPB5 and EPB22 (Pf1 + CHA0EP + B5 + EPB22). These plants showed a 60% reduction in the BBTV virus infection compared to untreated plants when grown in soil (Nowak and Shulaev, 2003).

Maksimov et al. (2019) showed that the isolates of *Pseudomonas* spp., which included *P. fluorescens*, *Pseudomonas putida*, *Pseudomonas aeruginosa*, *Pseudomonas taiwanensis*, and *Bacillus* spp., contributed to the protection of papaya plants against the ringspot virus (PRSV). According to studies by Maksimov et al. (2019), the preparation of Phytosporin-M based on *Bacillus subtilis* 26D also showed a high efficiency for plant protection against viral diseases, and the activity of these microorganisms was confirmed against a wide range of diseases, which included bacterial, fungal, and viral ones.

According to Maximov, the biocidal activity of rhizosphere and endophytic bacteria suggests that it indirectly exhibits antiviral activity due to their production of antibiotic substances. Hence, phytopathogenic bacteria, fungi, nematodes, and insect pests can be vectors-transporters of many plant viral infections. One promising approach to creating new antiviral drugs is to use strains of microorganisms with obvious insecticidal or other biocidal effects to control virus vectors. An isolate of *B. subtilis* BS3A25 and its culture filtrate inhibited the development of cucumber mosaic virus in tomato plants by inhibiting the development of the melon aphid *Aphis gossypii*, which is a vector of this disease that may be related to oxidation of surfactants that are produced by the bacteria (Rodríguez et al., 2018) and to the activation of the plant defense mechanisms against the insect and/or the virus that is under the influence of the bacteria (Maksimov et al., 2019). The colonization of internal onion tissues by the endophytic fungus *Hypocrea lixii* (F3ST1) significantly reduced the titer of iris yellow spot virus (IYSV) particles in plants, which reduced their damage by its main transmitter, which is called *Thrips tabaci* Lind (Muvea et al., 2018).

Bettoni et al. (2022) showed high effectiveness in regard to treating potatoes with arachidonic acid against *Phytophthora* and scab as well as against viruses X and M. It is promising to use preparations that are based on bacterial enzymes as elicitors. They became available after the success of genetic engineering in regard to creating highly productive strain producers. Diener (1961) established the antiviral activity of ribonucleases against RNA viruses and showed that the treatment of potato regenerants with RNase resulted in the inactivation of potato virus X and the tobacco mosaic virus in cucumbers.

Using elicitors to control viruses in agricultural plantings is a promising, affordable, and environmentally friendly method of viral disease control. Induced systemic resistance solves many problems with agricultural crop production. It is environmentally friendly and biosafe compared to biocides because it activates the natural plant’s defense mechanisms and is simultaneously effective against different phytopathogens under field conditions (Maksimov et al., 2019). The attractiveness of this direction is obvious because preparations with bioregulatory activity are used as immunomodulators, which induce the protective responses of the plant organism as well as positively affect the yield and quality indicators of plants. The search for virus inhibitors among the preparations with biological activity is of particular practical interest for potato producers because it will greatly simplify the task of obtaining healthy material for seed production (Acharya, 2013; Calil and Fontes, 2017; Somalraju et al., 2022). The proposed approaches for plant protection from phytoviruses are generalized in **Table 3**.

**Table 3:** The proposed approaches to plant protection from phytoviruses.

S. no.	Method	Source
1	Genome editing to combat virus infection	(Shan et al., 2013; Baltes et al., 2014; Price et al., 2015; Abudayyeh et al., 2017; Wolter and Puchta, 2018; Zhang et al., 2018; Zhan et al., 2019; Ahmad et al., 2020; Nussenzweig and Marraffini, 2020; Zhao et al., 2020; Nidhi et al., 2021; Hofvander et al., 2022; Kavuri et al., 2022; Robertson et al., 2022; Sharma et al., 2022; Silva and Fontes, 2022; Tiwari et al., 2022)
2	Apical meristem method	(Zhu-Jun et al., 2011; Hull, 2014; Anikina and Seitzhanova, 2015; Jin et al., 2016; Antonova et al., 2017; Galeev et al., 2018; Zhang et al., 2019)
3	Thermotherapy	(Wu et al., 2015; Oves and Gaytova, 2016; Wang et al., 2022)
4	Combination of thermotherapy methods and the method of apical meristems	(Wang et al., 2006; AlMaarri et al., 2012; Moses et al., 2017)
5	Cryotherapy	(Wang et al., 2006; Yi et al., 2014; Bi et al., 2018; Zhang et al., 2019)
6	Chemotherapy	(AlMaarri et al., 2012; Ryabtseva et al., 2015; Antonova et al., 2017; Yalovik et al., 2019; Bettoni et al., 2022)
7	Combination of thermotherapy and chemotherapy methods	(Fletcher et al., 1998; Yi Lan et al., 2005; Dhital et al., 2007; Nasir et al., 2010; Antonova et al., 2017)
8	Control of viruses vectors	(Jones, 2009; Hull, 2014; Calil and Fontes, 2017; Musidlak et al., 2017; Wei et al., 2018; Jones and Naidu, 2019; Batuman et al., 2020)
9	Creation of transgenic varieties with high resistance to viral diseases	(Rodríguez-Negrete et al., 2009; Sharipova et al., 2013; Wu et al., 2015; Hong and Ju, 2017; Kochetov and Shumny, 2017; Musidlak et al., 2017; Kourelis and van der Hoorn, 2018; Rodríguez et al., 2018; Tran et al., 2018; Yang and Li, 2018; Guo et al., 2019; Klimenko et al., 2019; Lolle et al., 2020; Akhter et al., 2021; Ross et al., 2021; Zhao et al., 2021; Bwalya et al., 2022; Li and Wang, 2022; Lopez-Gomollon and Baulcombe, 2022; Sáez et al., 2022; Liu et al., 2023)
10	Plant immunization	(Kothari and Patel, 2004; Dyakov et al., 2007; Calil and Fontes, 2017; Liu et al., 2017; Beris et al., 2018)
11	The use of biologically active preparations that suppress a viral infection	(French and Towers, 1992; Malhotra et al., 1996; Orazov and Nikitina, 2009; Mandal, 2010; Acharya, 2013; Alazem and Lin, 2017; Prasad and Srivastava, 2017; Maksimov et al., 2019; Chojnacka et al., 2021; Gupta AK. et al., 2021)

## 4 Conclusions and future perspectives

Viral infection in plants is a complex and interdependent process. Viruses are breeding programs to create virus-resistant forms based on genome editing, using the marker-mediated selection method, which makes it possible to evaluate the genetic protection of a variety and use RNA silencing as an important antiviral mechanism. The disadvantages of genetically engineered approaches include that the gained resistance is often specific and short-lived, and there is a problem with “gene silencing”. A negative factor of growing genetically changed plants resistant to certain viruses is the possible redistribution of virus species structure, which can provoke the spread of other harmful viral infections. The apical meristem method, combined with other auxiliary methods (thermotherapy and chemotherapy) during the last 50 years, has been the basis for controlling harmful plant viruses. Significant disadvantages of the practice of health improvement based on tissue culture include the possibility of auto-clonal variations because of long-term cultivation of plants under *in vitro* conditions, which are mutant plants genetically different from the original variety. In addition, this technique does not guarantee against rapid reinfection of diseased material under field conditions.

The use of virus inhibitors and elicitors is most justified in the field. At present, several groups of synthetic antiviral compounds have been identified. The efficacy of virocides has been proven in outdoor plant treatments. A limiting factor is the phytotoxicity of virocides, and also the teratogenic effect of some virocides concerning humans has been revealed. Using elicitors to control viruses in agricultural plantings is a promising, affordable, and environmentally friendly approach to preventing viral diseases. Induced systemic resistance is environmentally friendly and biosafe when compared to biocides because it activates natural plant defense mechanisms and is effective simultaneously against different phytopathogens under field conditions. The disadvantages of the method include insufficiently high efficiency. Vector control is a well-known method for preventing the spread of viral infection. The limitations, in this case, are low efficiency and high costs. In addition, considerable damage has been inflicted to the ecology. An alternative direction is the agroecosystem approach based on the study of aspects of multitrophic interactions where the activity of microbial strains with clear insecticidal or other biocidal effects is used to control vector-borne viruses. The antiviral effectiveness of drugs based on microbial enzymes has also been shown. The disadvantages of using microbial preparations include low efficacy. As shown in the literature review, the existing methods of phytovirus control are ambiguous. Further research is needed into plant resistance mechanisms, including the molecular interaction between the host and viral factors. Further study of genetic, biochemical, and physiological features of viral pathogenesis and the development of a strategy for increasing plant resistance to viruses will allow a new level of control of phytovirus infection to be achieved.

## Author contributions

All authors listed have made a substantial, direct, and intellectual contribution to the work and approved it for publication.
